# Linking Carbon Fluxes to Flooding Gradients in Sediments of Mediterranean Wetlands

**DOI:** 10.1021/acsestwater.4c00940

**Published:** 2025-05-06

**Authors:** Carlos Rochera, Antonio Picazo, Daniel Morant, Javier Miralles-Lorenzo, Vanessa Sánchez-Ortega, Antonio Camacho

**Affiliations:** a Cavanilles Institute of Biodiversity and Evolutionary Biology, 16781 University of Valencia, C/Catedrático José Beltrán, 2, Paterna, Valencia E-46980, Spain; b Fundación Global Nature, C/Tajo, 2, Las Rozas de Madrid E-28231, Spain

**Keywords:** Mediterranean wetlands, wetland management and restoration, wetland sediment processes, methane production regulation, flooding regimes, climate change mitigation

## Abstract

This study examines the seasonal variability of greenhouse gas (GHG) emissions from wetland sediments in the Iberian Peninsula in relation to water levels. It included coastal marshes, inland freshwater, and inland saline wetlands, three typical regional types. GHG fluxes peaked in coastal wetlands and were lowest in saline ones. Flux variations were driven by water depth, salinity, and sediment aeration. CO_2_ emissions peaked in dry zones and declined with water depth, while CH_4_ fluxes were more variable in waterlogged transition zones, particularly in coastal wetlands during spring and summer. CH_4_ emissions were lower in well-aerated, less-flooded areas and highest in shallowly flooded zones, where even a thin water layer restricts gas exchange, limiting oxygen and maintaining anaerobic conditions for methanogenesis. However, the lack of a deep-water column prevented methane oxidation, allowing diffusion into the atmosphere. Seasonal variation was higher in saline wetlands due to drought, while patterns in freshwater and coastal wetlands remained spatially more stable. Understanding these gradients is crucial for accurately modeling gas exchanges and assessing their role in climate change mitigation and adaptation. As interest in wetland carbon dynamics increases, integrating this modeling into management is vital to support restoration and long-term wetland sustainability.

## Graphical abstract

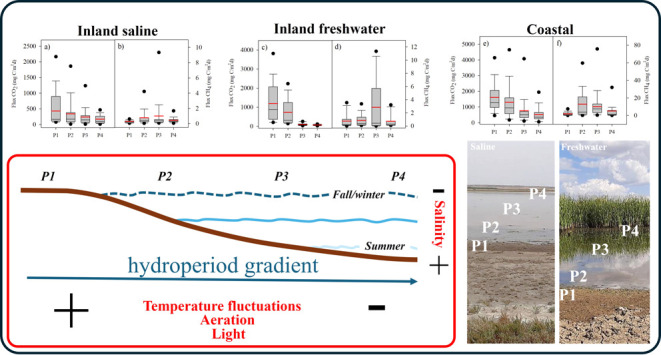

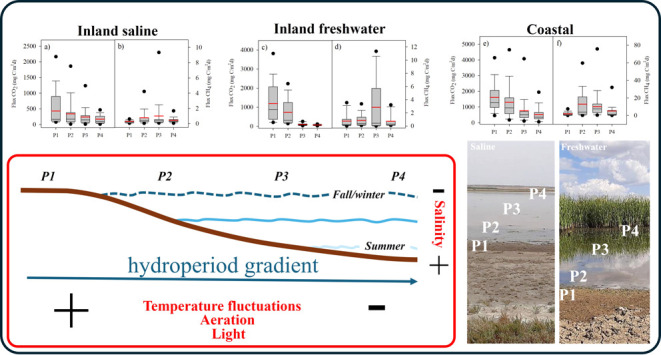

## 1 Introduction

Wetlands are vital in mitigating greenhouse gas (GHG) emissions by naturally sequestering carbon and minimizing overall emissions.^1^ The extent of these benefits depends on specific wetland features and how they are managed. Water level fluctuations play a crucial role in the ecological functioning of wetlands, influencing both their management and restoration strategies.^2,3^ In Mediterranean wetlands, where seasonal hydrological patterns are prevalent, greenhouse gas (GHG) fluxes—particularly carbon dioxide (CO_2_) and methane (CH_4_)—vary significantly in response to these changes. These fluctuations impact carbon dynamics, shifting the balance between CO_2_ and CH_4_ emissions as different metabolic processes dominate under varying water and oxygen conditions.^4^ For example, periods of flooding can enhance anaerobic conditions, favoring methanogenesis and thus increasing CH_4_ emissions, while drier conditions often promote aerobic decomposition, leading to higher CO_2_ release.

Due to the shallow nature of these ecosystems, both diffusion and ebullition are key pathways for CH_4_ transport, contributing substantially to total emissions. Ebullition, in particular, can account for a large portion of CH_4_ emissions, with estimates ranging from 18 to 50% of total CH_4_ output in wetlands.^5^ The interaction between water levels and gas exchange is complex, as fluctuating water levels not only regulate the extent of anaerobic zones but also influence the frequency and intensity of ebullitive events. Furthermore, the dynamics of GHG emissions are closely tied to water level variations, which impact the rates of diffusion and ebullition, resulting in seasonal and environmental variations in emission rates.^6^

This study focuses on three main wetland types in the Iberian Peninsula: inland nonsaline, inland saline, and coastal marshes. Each type has distinct hydrological characteristics, from the regulated hydrology of nonsaline wetlands to the unpredictable, weather-dependent saline systems, and the managed, highly productive coastal marshes. These differing hydrological regimes drive variability in GHG emissions, affecting both the intensity and distribution of fluxes throughout the year. GHG measurements were obtained from sediment cores at various flood levels to capture these spatial and temporal variations in emissions, providing a comprehensive assessment of how water dynamics shape carbon fluxes in these ecosystems. The goal is to provide insights that will inform management strategies aimed at reducing GHG emissions while preserving the ecological integrity and functions of these critical wetland habitats.

This study aims to analyze the influence of water level fluctuations on greenhouse gas (GHG) emissions in various wetland types of the Iberian Peninsula. By examining in diverse types of Mediterranean wetlands, we aim to capture the spatial and seasonal variations in CO_2_ and CH_4_ emissions along a flooding gradient. This research provides a comprehensive assessment of how water dynamics shape carbon fluxes, exploring how factors such as main physicochemical factors related with water depth, salinity, and oxygenation interact to drive these processes. The findings will inform wetland management strategies focused on reducing GHG emissions while preserving ecological integrity. Ultimately, this study contributes to a more nuanced understanding of wetlands’ role in climate change mitigation, highlighting the need for targeted management and restoration practices that account for the complexity and variability of these ecosystems.

## 2 Material and Methods

### 2.1 Study Site

A total of 10 waterbodies representative of main wetland types within Iberian Peninsula were selected for the study (Figure 1). The inland nonsaline wetlands studied—Laguna de La Nava and Laguna de Boada—are located in the Tierra de Campos region (Palencia, Castilla y León, Spain), within the northwestern Mediterranean biogeographical zone of the Iberian Peninsula. Both sites have regulated hydroperiods, receiving water through channels that flood the areas from early autumn to early summer. Laguna de Boada consists of a single basin where water enters from the northern region, while Laguna de La Nava has a more complex system of channels and basins, including a permanent section that retains water year-round. The southwestern part of La Nava, separated by a road, is managed differently, with flooding based on local farmers’ grazing needs.

**Figure 1:**
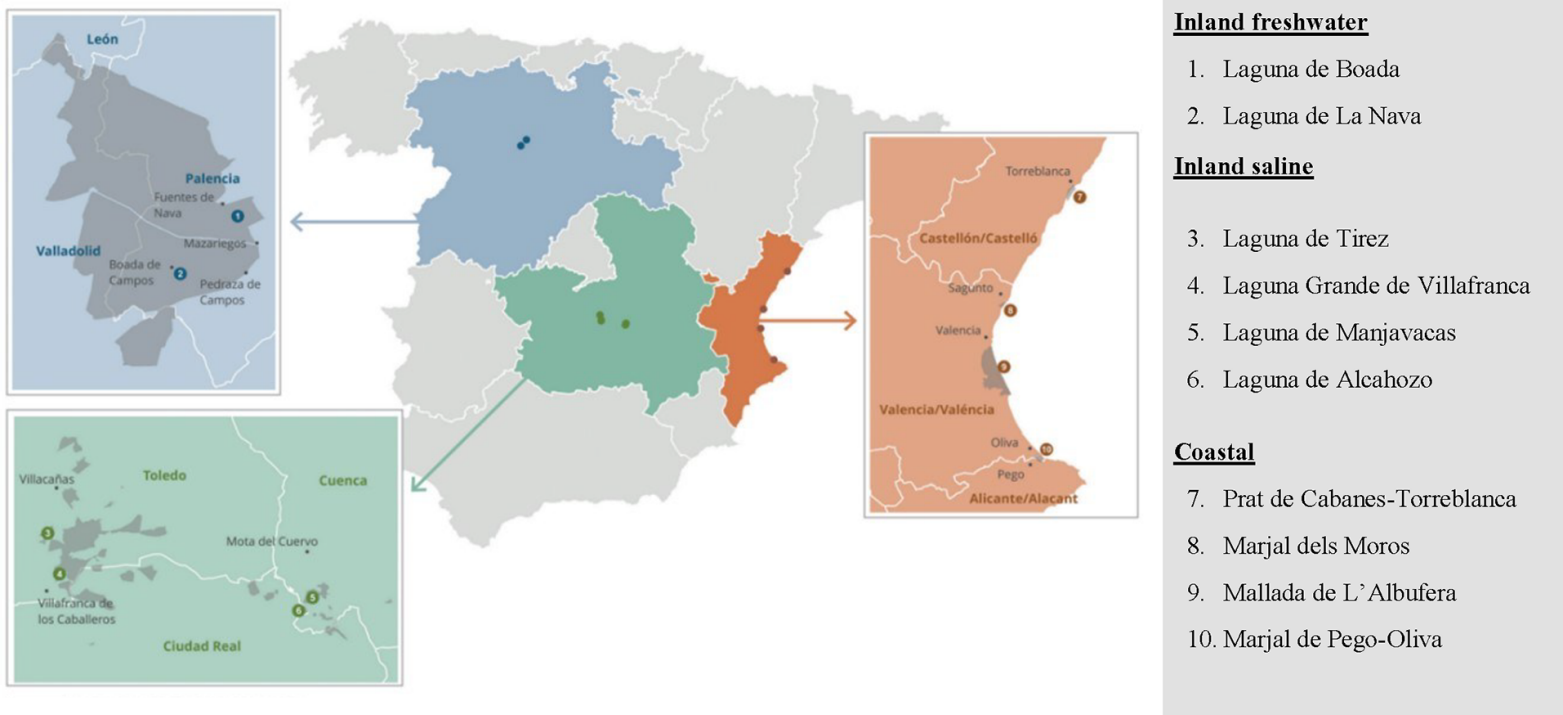
Geographic location of the studied wetlands across Spain. The sites are categorized into three types: inland freshwater (Laguna de Boada and Laguna de La Nava), inland saline (Laguna de Tirez, Laguna Grande de Villafranca, Laguna de Manjavacas, and Laguna de Alcahozo), and coastal wetlands (Prat de Cabanes-Torreblanca, Marjal dels Moros, Mallada de l’Albufera, and Marjal de Pego-Oliva).

The four inland saline wetlands analyzed are located within the UNESCO La Mancha Húmeda Biosphere Reserve in central Spain —Laguna de Tirez, Laguna de Alcahozo, Laguna de Manjvacas and Laguna Grande de Villafranca.^7^ These saline steppe lakes, characterized by pronounced seasonality, display significant intra- and interannual variability, driven primarily by meteorological conditions.^8^ Laguna de Alcahozo and Laguna de Tirez exemplify this pattern, with Tirez becoming increasingly ephemeral due to rising aridity and water scarcity, flooding only during periods of heavy rainfall. In contrast, Laguna de Manjavacas and Laguna Grande de Villafranca have altered hydrological regimes that extend their hydroperiods. Laguna de Manjavacas receives inflows from a nearby wastewater treatment plant, reducing its salinity and making it semipermanent, while Laguna Grande de Villafranca remains permanent due to inflows from the Cigüela River, serving as a public recreational area.

The four coastal marshes studied — Marjal de Pego-Oliva, Prat de Cabanes-Torreblanca, Marjal dels Moros, and Mallada de La Albufera — are highly regulated ecosystems that have undergone significant hydro-morphological changes over recent decades. Formerly used as rice paddies, many areas have been restored to wetlands through abandonment or active intervention, creating interconnected, floodable parcels linked by canals. Ongoing management and human activities have further increased the anthropogenic impact on these ecosystems.

### 2.2 Measurement of In Situ Physical–Chemical Characteristics

Temperature, conductivity, salinity, dissolved oxygen, and pH were measured in situ at representative wetland locations using a WTW multiparameter probe with calibrated sensors. Dissolved oxygen concentrations were corrected for salinity effects to ensure accurate readings.

### 2.3 Survey Design and Sediment Cores Sampling

Greenhouse gas (GHG) exchanges were evaluated using the undisturbed sediment core method.^7,9,10^ Four sampling points were selected across the flood gradient within the lagoon basin, covering a range from the outer, typically drier areas to the inner, deeper zones that experience more frequent inundation (Figure 2). The natural inundation status of each site was strictly maintained inside the cores, meaning that sediment cores reflected the actual water column height found in the field at the time of collection. This meant, for instance, that in temporary wetlands such as Tirez or Alcahozo, all sampling points were dry during certain sampling events, particularly in the warmer months, and were analyzed accordingly. Following this procedure, both dry and inundated conditions were naturally integrated into the study. The sampling set up consisted in dividing the basin into four distinct zones along a flooding gradient to establish four sampling points. The outermost dry zone (P1) represents areas that are typically dry or have minimal water during summer. Adjacent to this is the shallow wet zone (P2), which may experience intermittent flooding during the wet season. Moving inward, the shallower waters are consistently shallow zones that remain inundated but are not deeply submerged (P3). Finally, the deeper waters comprise the innermost zone with the highest water levels, remaining inundated throughout most of the year (P4).

**Figure 2:**
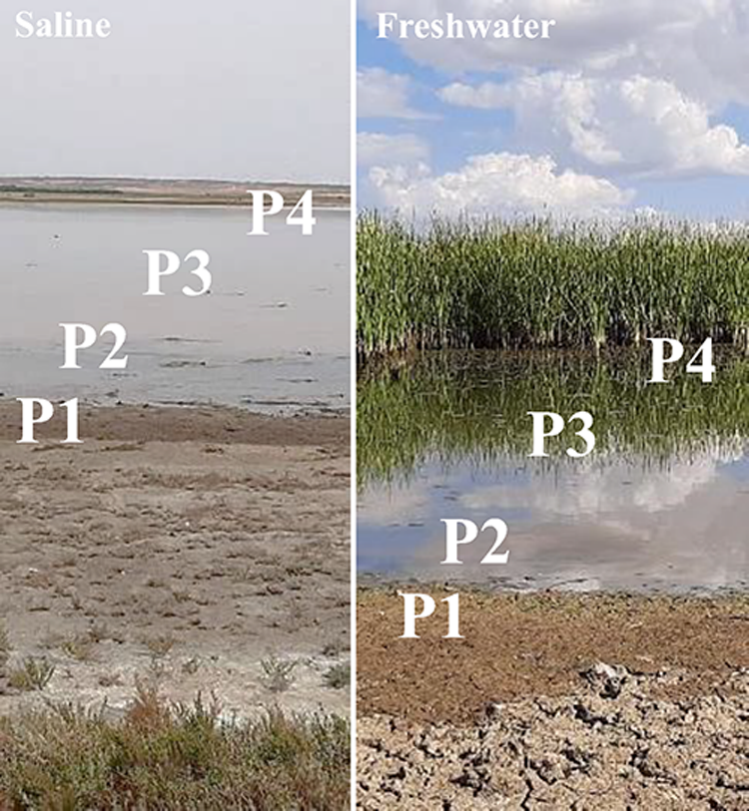
Pictures showing the flooding gradient in both saline (left) and freshwater (right) wetlands. The zones (P1 to P4) represent different hydrological conditions: P1 corresponds to the dry outermost area, P2 to the intermittently flooded zone, P3 to the consistently shallow water zone, and P4 to the deeper, permanently inundated area. This gradient highlights the variation in wetland water coverage, influencing carbon fluxes and ecological processes.

Once the sampling points were determined, transparent methacrylate tubes, measuring 50–75 cm in length and 4 cm in diameter, were used to extract sediment cores. Six replicate cores, each approximately 15 cm in length, were collected for each experimental condition. Tubes were filled with lake water to replicate actual inundation conditions and least 15 cm of air was maintained at the top to create a headspace. The tubes were then sealed at the bottom with airtight plastic lids,, and transported upright to the laboratory. Transport to the laboratory took less than 3 h, with the cores kept at ambient temperature and experiencing only minor thermal variation, as the sampling and incubation sites were situated in locations with comparable climatic conditions.

### 2.4 Measurement of Carbon Fluxes

In the lab, the cores were placed inside a climatic chamber, where the temperature was set to replicate in situ conditions. To minimize temperature fluctuations, the cores were positioned within a water-filled container, ensuring additional thermal stability. Under this setup, the water column and headspace of the cores were directly exposed to natural light. Given the shallow water depth in the inundated cores, it was assumed that light penetration was sufficient to support photochemical and microbial processes similar to those occurring in the field.

Before incubation, headspace air from open cores was pumped through the LICOR LI-7810 gas analyzer until CO_2_ and CH_4_ concentrations equilibrated with ambient levels, which were recorded as the initial conditions for incubation. The tubes were then sealed with airtight lids and placed back in the climatic chamber under natural light and temperature conditions. A short incubation period of 1–2 days was used, depending on the expected carbon fluxes, to capture greenhouse gas emissions that closely reflect near in situ conditions at the time of sampling.

To determine CO_2_ and CH_4_ concentrations at the end of the incubation period, small headspace volumes (0.2–0.5 mL) from cores were injected through an open-loop system into the gas analyzer, following the protocol described by LI-COR Biosciences.^11^ As per the manufacturer’s specifications, this gas analyzer requires a nonzero reference measurement of the target gases for proper operation. Accordingly, particle-free ambient air, delivered through an air compressed system, was used as carrier gas to maintain stable reference conditions. The CO_2_ and CH_4_ levels in this carrier air remained at typical atmospheric concentrations, and sample injections into the open-loop system were recorded as variations relative to this baseline.

The concentration of triplicated injected samples was determined by measuring the area under the curve generated immediately after be injected into the gas analyzer through a 3-way compression fitting (LICOR, P/N 9881–181), as described by LI-COR Biosciences.^11^ The concentration was then calculated based on the injected volume and the empirical relationship between area and gas concentration, which was established through previously generated calibration curves using known gas concentrations.

The CO_2_ and CH_4_ measurements recorded by the LICOR LI-7810 gas analyzer were in ppm and ppb, respectively, and the surface emission rates were calculated in mg C/m^2^·day using the Ideal Gas Law. The differences between the initial and final gas concentrations in the headspace represented the accumulated gas over a known surface area, volume, and time period.

Statistical differences between treatment and season groups were assessed using the Kruskal–Wallis test, as data did not generally meet normality and homogeneity of variance assumptions. Statistical significance was set at *p* < 0.05. Both the Kruskal–Wallis H statistic and the associated p-value are provided throughout the text.

## 3 Results

### 3.1 Overall Patterns of CO_2_ and CH_4_ Fluxes

The fluxes of CO_2_ and CH_4_ were generally higher on average in coastal wetlands, followed by freshwater wetlands, while the lowest average fluxes were observed in saline wetlands (Figure 3). In all wetland types, the mean CO_2_ emissions, represented by the red lines, were highest in the dry, outermost zone (P1).

**Figure 3:**
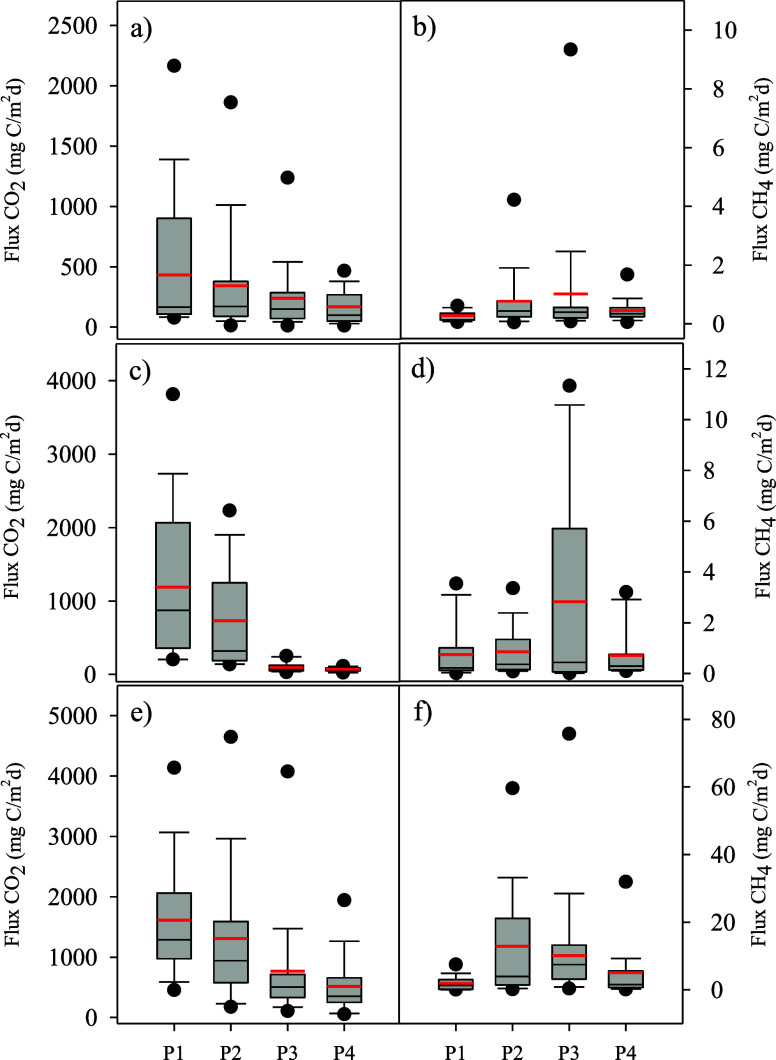
CO_2_ (left panels: a, c, e) and CH_4_ (right panels: b, d, f) fluxes across different flooding zones (P1–P4) in three types of wetlands: saline (a, b), freshwater (c, d), and coastal (e, f). P1 represents the dry outermost zone, while P4 corresponds to the innermost and deepest water zone. Box plots illustrate the variability in fluxes, with red lines indicating mean values.

In saline wetlands, mean CO_2_ fluxes in P1 were below 500 mg C/m^2^/day (Figure 3a), while freshwater wetlands averaged slightly over 1,200 mg C/m^2^/day (Figure 3b), and coastal wetlands showed means around 1,500 mg C/m^2^/day (Figure 3). This indicates that drier conditions favor aerobic metabolism and promote CO_2_ release.

As water levels increased from P2 to P4, the mean CO_2_ fluxes decreased across all wetland types. This decrease was particularly pronounced in the inland freshwater wetlands, with mean fluxes dropping significantly in the shallow and deep-water zones (Figure 3). In contrast, saline and coastal wetlands showed a less marked decrease, with mean CO_2_ fluxes in the wetter zones (P3 and P4), averaging between 200 to 800 mg C/m^2^/day.

For CH_4_ fluxes, the mean values in the transition zone from waterlogged (P2) to shallow water (P3) showed considerable dispersion, reflecting seasonal changes in water levels and environmental conditions, especially affecting coastal and freshwater ecosystems. Saline wetlands exhibited much lower and more stable mean CH_4_ fluxes across P2 and P3 (Figure 3b). Freshwater wetlands had mean CH_4_ fluxes around 2.5 mg C/m^2^/day in P3, while coastal wetlands showed averages of slightly above 10 mg C/m^2^/day (Figure 3f).

### 3.2 Seasonal Patterns of Carbon Fluxes in the Inland Saline Wetlands

In both the lagoon of Tirez and the lagoon of Alcahozo, the most temporary and hydrologically stressed wetlands in the study, CO_2_ and CH_4_ fluxes fluctuated rather than showing a consistent decline with depth, unlike the typical pattern in other wetlands (Figure 4).

**Figure 4:**
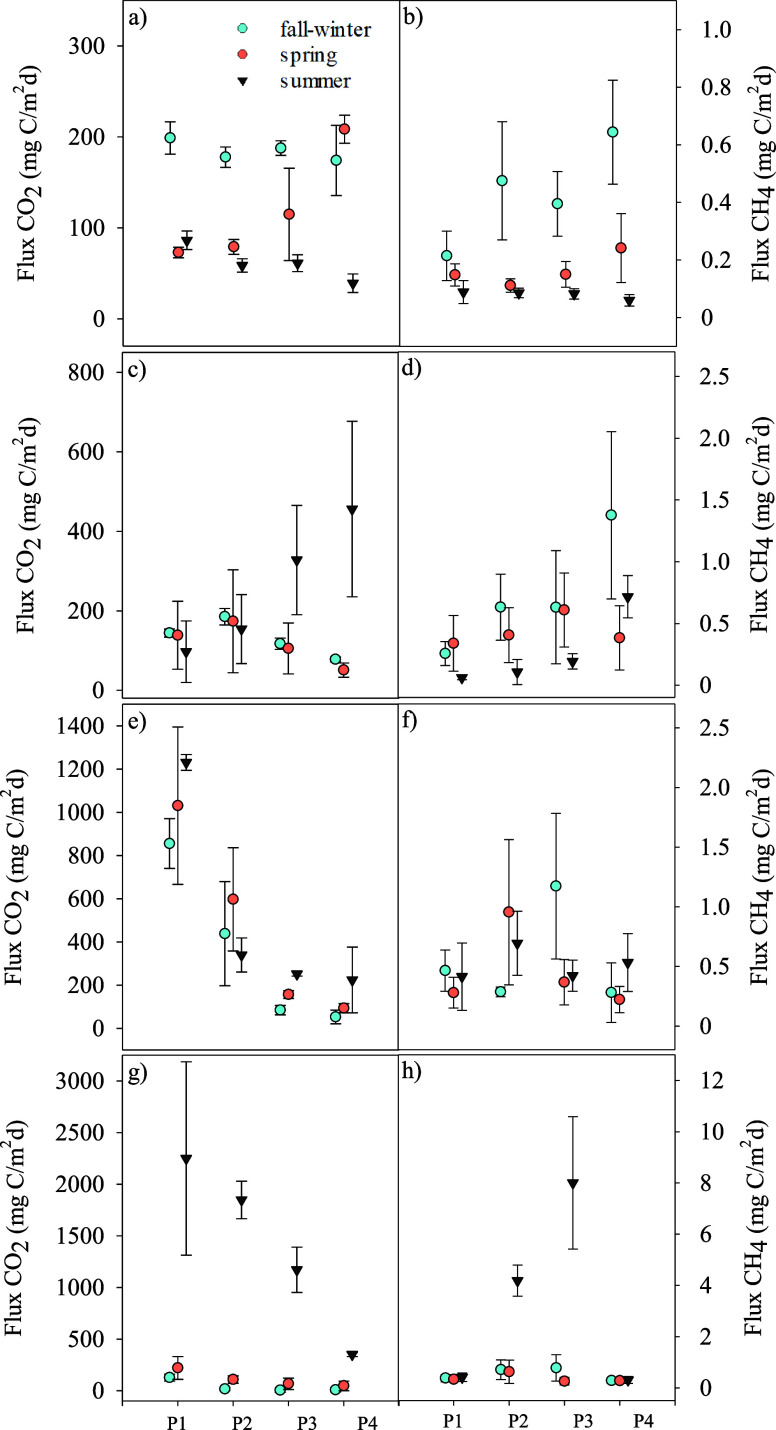
Seasonal variations in CO_2_ (left panels: a, c, e, g) and CH_4_ (right panels: b, d, f, h) fluxes across the flooding gradient in inland saline wetlands. The *x*-axis represents the gradient zones (P1: outermost dry zone, P2: shallow wet zone, P3: shallower waters, P4: deeper waters). Panels (a) and (b) correspond to Laguna de Tirez, (c) and (d) to Laguna de Alcahozo, (e) and (f) to Laguna de Grande Villafranca, and (g) and (h) to Laguna de Manjavacas. Symbols indicate different seasons: fall-winter (cyan circles), spring (orange circles), and summer (black triangles). Error bars represent the standard deviation of flux measurements.

In Tirez, which is frequently dry, CO_2_ flux generally increased from P1 (dry zone) to P4 (deeper waters). Significant differences were found (H = 20.67, *p* < 0.001), with the highest fluxes occurring in fall-winter, often exceeding 200 mg C/m^2^/day, while spring showed moderate values (100–200 mg C/m^2^/day). Summer had the lowest fluxes, remaining below 100 mg C/m^2^/day due to the total drought of the soil (Figure 4a). Similarly, CH_4_ fluxes increased from P1 to P4, with also significant (H = 26.61, *p* < 0.001) highest values (0.4–0.6 mg C/m^2^/day) recorded in fall-winter, while spring had moderate fluxes, and summer displayed the lowest emissions (Figure 4b).

In Alcahozo, which is seasonal but less dry than Tirez, CO_2_ fluxes fluctuated rather than declining with depth (Figure 4c). The highest fluxes were observed in summer, particularly in P3 and P4, reaching around 400 mg C/m^2^/day, although differences between seasons did not reach statistical significance when considered all points of the gradient (H = 5.73, p = 0.057). Fall-winter and spring showed more consistent fluxes across zones, between 100 and 200 mg C/m^2^/day. CH_4_ fluxes also increased from P1 to P4, with fall-winter showing the highest values, around 2.0 mg C/m^2^/day in P4. Spring and summer had significant (H = 8.27, p = 0.016) lower CH_4_ emissions, mostly between 0.2 and 1.0 mg C/m^2^/day (Figure 4d).

In contrast, the lagoon of Grande de Villafranca, with a longer hydroperiod, followed the typical wetland pattern. CO_2_ fluxes decreased from P1 to P4, with the highest values in P1, averaging 800–1,200 mg C/m^2^/day, and dropping below 400 mg C/m^2^/day in deeper zones (Figure 4e). CH_4_ fluxes increased moderately in the water-saturated transition zone (P2–P3), with the highest fluxes during fall-winter and spring, while summer showed lower emissions (Figure 4f). Nevertheless, no significant statistical differences were observed between seasons for both CO_2_ (H = 0.34, p = 0.843) and CH_4_ (H = 2.17, p = 0.339) fluxes.

Finally, in Manjavacas, where eutrophication and an artificially prolonged hydroperiod due to wastewater input affect the dynamics, CO_2_ fluxes peaked significantly (H = 26.81, *p* < 0.001) in summer, exceeding 2,000 mg C/m^2^/day in P1­(Figure 4g). CO_2_ emissions remained low during other seasons, generally below 200 mg C/m^2^/day. CH_4_ fluxes were highest in the transition from P2 to P3, reaching around 8 mg C/m^2^/day during summer, when P3 stayed waterlogged (Figure 4h).

### 3.3 Seasonal Patterns of Carbon Fluxes in the Inland Freshwater Wetlands

In Laguna de Boada, CO_2_ fluxes consistently decreased from P1 (dry zone) to P4 (deeper waters). The outermost zone (P1) had the highest mean fluxes, particularly in spring (Figure 5a), reaching around 1,200 mg C/m^2^/day and being significantly (H = 7.05, p = 0.03) higher compared to the other periods. As depth increased toward P4, CO_2_ emissions dropped significantly, with values generally falling below 200 mg C/m^2^/day. Meanwhile, CH_4_ fluxes did not follow a clear depth pattern but fluctuated slightly across zones (Figure 5b). The most notable emissions occurred in the water-saturated zones (P2 and P3) during spring, which as observed for CO_2_, was the period showing significantly higher emissions (H = 13.67, p = 0.001). In this season, fluxes at these water-saturated zones peaked at around 0.8 mg C/m^2^/day, while in P4, CH_4_ fluxes remained below 0.5 mg C/m^2^/day.

**Figure 5:**
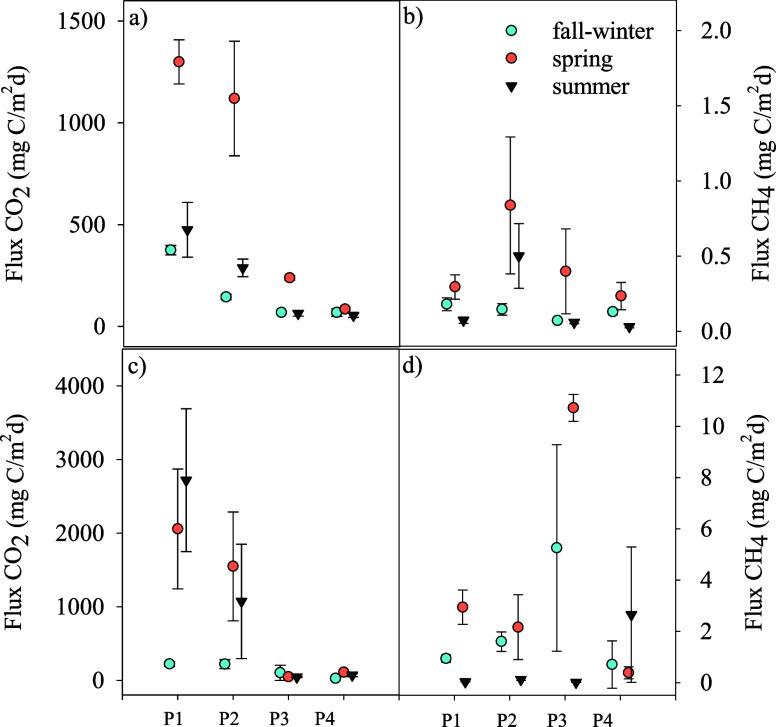
Seasonal variations in CO_2_ (left panels: a, c) and CH_4_ (right panels: b, d) fluxes across the flooding gradient in inland freshwater wetlands. The *x*-axis represents the gradient zones (P1: outermost dry zone, P2: shallow wet zone, P3: shallower waters, P4: deeper waters). Panels (a) and (b) correspond to Laguna de Boada, while panels (c) and (d) represent Laguna de La Nava. Symbols indicate different seasons: fall-winter (cyan circles), spring (orange circles), and summer (black triangles). Error bars show the standard deviation of flux measurements.

Laguna de La Nava also showed also a decline in CO_2_ fluxes from P1 to P4, although with substantial emissions recorded in both spring and summer (Figure 5c). The highest mean values were around 2,000 and 2,700 mg C/m^2^/day, respectively. CH_4_ fluxes displayed an increasing trend from P1 to P3 (Figure 5d), peaking in the intermediate, water-saturated zone (P3) during spring and fall-winter, reaching up to 10 mg C/m^2^/day. In the deeper zone (P4), CH_4_ emissions decreased, often staying below 2 mg C/m^2^/day. The most significant CH_4_ emissions were noted in P3 during spring and fall-winter, although only spring showed a significant difference (H = 11.74, p = 0.003) due to high data dispersion. On the other hand, summer generally exhibited lower emissions across all zones.

### 3.4 Seasonal Patterns of Carbon Fluxes in the Coastal Wetlands

The CO_2_ and CH_4_ fluxes in coastal wetlands demonstrated patterns influenced by their higher productivity and hydrological stability compared to previous wetland types.

In Marjal de Pego-Oliva, CO_2_ fluxes declined slightly from P1 to P4 but generally remained moderate (Figure 6a). The highest emissions occurred in the outer zones (P1 and P2), particularly during spring and fall-winter, reaching values around 1,300 mg C/m^2^/day. However, no significant differences were observed between seasons when all points of gradient were considered together (H = 1.92, p = 0.383). As depth increased toward P4, fluxes dropped below 500 mg C/m^2^/day. As shown in Figure 6b, the CH_4_ fluxes peaked significantly (H = 12.99, p = 0.002) during spring in the intermediate zones (P2 and P3), averaging around 25 mg C/m^2^/day, while the outermost (P1) and deeper (P4) zones exhibited lower emissions, typically below 5 mg C/m^2^/day.

**Figure 6:**
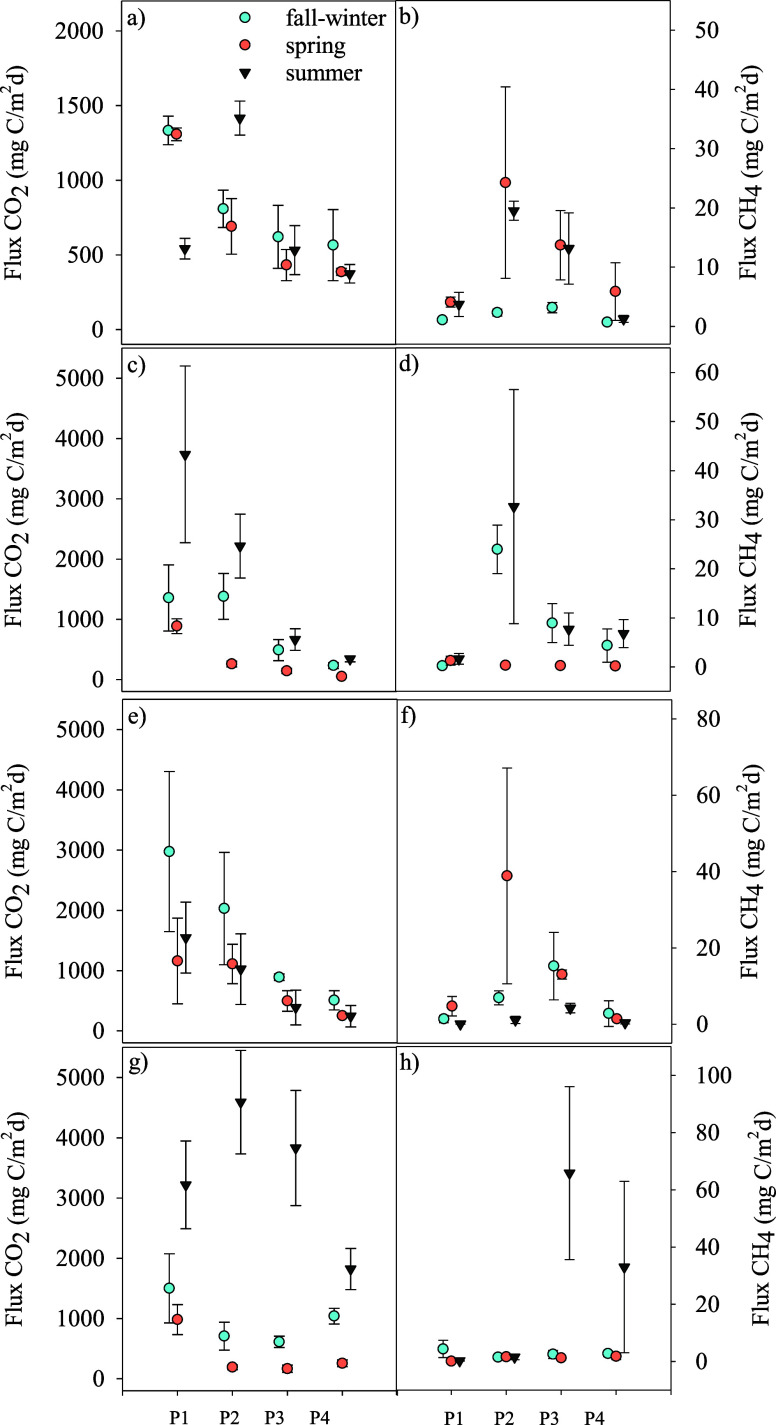
Seasonal variations in CO_2_ (left panels: a, c, e, g) and CH_4_ (right panels: b, d, f, h) fluxes across the flooding gradient in coastal wetlands. The *x*-axis represents the gradient zones (P1: outermost dry zone, P2: shallow wet zone, P3: shallower waters, P4: deeper waters). Each row corresponds to a different coastal wetland: (a-b) Marjal de Pego-Oliva, (c-d) Prat de Cabanes-Torreblanca, (e-f) Marjal dels Moros, and (g-h) La Mallada de la Albufera. Symbols indicate different seasons: fall-winter (cyan circles), spring (orange circles), and summer (black triangles). Error bars represent the standard deviation of flux measurements.

In Prat de Cabanes-Torreblanca, a similar decline in CO_2_ fluxes along gradient was observed (Figure 6c), with values falling below 500 mg C/m^2^/day in the deeper zones. This wetland showed highest CO_2_ (H = 12.01, p = 0.002) and CH_4_ (H = 15.65, *p* < 0.001) emissions during summer as shown in Figure 6c and 6d, respectively, while fall-winter and spring presented lower, more stable values across the gradient.

Marjal dels Moros followed a comparable pattern, with CO_2_ fluxes decreasing along the flooding gradient (Figure 6e). Conversely to Prat de Cabanes-Torreblanca, the peak occurred during the fall-winter filling period rather than in summer, although without significant differences between periods (H = 4.07, p = 0.131). CH_4_ fluxes peaked in the transition zones (P2 and P3), particularly during spring (Figure 6f), averaging around 40 mg C/m^2^/day and being significantly higher compared to the other periods (H = 12.13, p = 0.002).

In the interdunal coastal wetland of La Mallada de la Albufera, the sandy soils facilitated gas exchange, significantly shaping the flux patterns and making they higher compared to the other coastal wetlands. CO_2_ fluxes were highest during summer (H = 26.21, *p* < 0.001), peaking in P2 and remaining elevated toward the innermost parts of the gradient (Figure 6g). On the other hand, fall-winter and spring displayed lower and more stable fluxes across all zones. CH_4_ emissions in this interdunal coastal wetland peaked in the intermediate shallow zone (P3) during summer (Figure 6h), averaging around 66 mg C/m^2^/day, with slightly lower values in P4. However, differences between seasons did not reach statistical significance (H = 5.85, p = 0.054) due to high data dispersion.

## 4 Discussion

The flooding gradient effectively represents varying levels of water coverage within wetlands, where the outermost zones experience infrequent flooding, and the innermost zones are more likely to remain submerged. By examining these gradients, our study captures the inherent heterogeneity of wetland ecosystems, providing detailed, localized data.

In this context, P1 corresponds to the outermost area, typically dry or with minimal water during summer. P2 usually represents a waterlogged zone that undergoes intermittent flooding during the wet season. Conversely, P3 lies in consistently shallow water zones that remain inundated but are not deeply submerged, while P4 is the innermost zone with the highest water levels, remaining inundated for most of the year. As water levels fluctuate seasonally, the shallower areas (P1 and P2) experience greater temperature variations, higher aeration, and more light penetration. Meanwhile, the deeper areas (P3 and P4) provide more stable conditions, although they may still experience partial or total drought during the summer in more temporary wetlands.

Our results consistently indicate that water presence along this gradient strongly regulates carbon fluxes across all wetland types. CO_2_ emissions peak in dry or intermittently flooded zones, then significantly decrease with increased water saturation. This inverse relationship suggests that higher soil saturation effectively reduces CO_2_ emissions. In contrast, CH_4_ emissions display the opposite pattern, increasing as conditions become more saturated. Peak CH_4_ emissions occur in waterlogged or shallowly inundated areas, while both dry and deeply flooded zones exhibit lower emissions. It should be noted that the flux estimations performed in this study account for both diffusive and ebullitive CH_4_ emissions, as the incubation period (1–2 days) was sufficiently long to allow both processes to occur at their natural rates. This approach avoided potential biases associated with short-term sampling, which may either overestimate ebullition due to gas handling disturbances or underestimate it by missing episodic bubble release.

In addition to capturing both emission pathways, the short incubation approach adopted in this study was intended to capture greenhouse gas fluxes more closely associated with in situ conditions. Similar short-term incubations—typically lasting from several hours to a few days—have been successfully employed in comparable studies to estimate CO_2_ and CH_4_ emissions from wetland sediment cores (e.g.,^12,13^ ). We assume that these shorter durations minimize experimental artifacts, such as oxygen depletion or shifts in microbial communities, and help avoid artificially extending microbial dynamics beyond field-representative conditions.

Our approach contrasts with studies designed to track long-term sediment stabilization through repeated or prolonged incubations (e.g.,^14,15^ ), where incubation systems are periodically opened to allow measurements. While this may help prevent cumulative changes, it also introduces variability. Moreover, such approaches often involve modifying experimental conditions—such as refreshing or replacing overlying water—which we deliberately avoided to preserve the integrity of the original sediment–water environment.

According to the patterns described for CO_2_ and CH_4_ in the present study, global warming potential (GWP) fluxes rise as water levels decline, particularly in zones that are either typically dry or transition between wet and dry conditions. Seasonal dynamics further influence these emissions; methane production increases during warmer periods due to its temperature sensitivity and intermediate water levels that promote methanogenesis. Indeed, methane fluxes exhibit a strong dependence on temperature, rising substantially during warmer periods as a result of increased methanogenic activity.^16^

Our study aligns with previous research demonstrating that methane (CH_4_) emissions are significantly reduced when water depth surpasses certain threshold values. For example, research has shown that a water depth of 50 cm is a critical threshold beyond which CH_4_ emissions decline substantially.^15^ This phenomenon occurs because deeper water levels limit the exchange of gases between the soil and atmosphere, thereby reducing methane flux. Additionally, the oxygenated upper water layer in deeper zones inhibits methanogenic archaea—the microorganisms responsible for methane production—further lowering CH_4_ emissions.

In inland wetlands, both saline and nonsaline, CH_4_ production was generally low to moderate, indicating that CO_2_ plays a more dominant role in the overall GWP balance. For instance, methane emissions in inland saline lagoons were found to be one to 2 orders of magnitude lower than those typically observed in comparable freshwater systems. This disparity likely arises because saline conditions inhibit methane fluxes by suppressing the activity of methanogenic bacteria, which thrive more efficiently in freshwater environments.^7^

While saline wetlands exhibit lower metabolic rates and, consequently, more moderate gas fluxes, their overall warming potential is significantly reduced. Nonetheless, similar patterns emerge as in other wetlands, with CO_2_ emissions peaking in nonflooded areas and methane emissions maximizing at the interface between saturated and low-water zones.

However, in specific wetlands like the lagoons of Tirez and Alcahozo, CO_2_ and CH_4_ fluxes deviated from the usual pattern. Here, rather than a consistent decline with depth, these fluxes showed fluctuations. This atypical pattern is likely due to the high hydric stress in the outer, dry zones of these temporary lagoons, which can become so extreme that it inhibits microbial activity, consequently reducing gas emissions.

In coastal marshes, such as those in southwestern Spain (e.g., Doñana), methane emissions exhibit significant spatial and temporal variability. These emissions are influenced by water salinity and temperature dynamics, which, in turn, affect sedimentary methanogenesis. CH_4_ concentrations vary widely depending on environmental conditions and primary productivity, indicating that methane dynamics are controlled by a complex mosaic of environmental processes.^17^

The gradient-based approach utilized in this study reduces errors often associated with large-scale extrapolations, which can lead to inaccuracies in regional and global emission estimates. By avoiding broad generalizations, our data provide a more detailed assessment of emission patterns across different wetland zones, offering a higher resolution of greenhouse gas (GHG) fluxes. For instance, methane (CH_4_) fluxes in wetlands are highly sensitive to small-scale environmental variations, such as temperature and water table depth. These localized differences significantly influence methane emissions, leading to substantial variability across different patches within the same wetland.^18^

Moreover, the observed variability in GHG emissions throughout the year underscores the importance of accounting for temporal changes when assessing wetland emissions. Studies have shown that CH_4_ and CO_2_ emissions vary significantly with seasonal temperature fluctuations, generally peaking during the warmer summer months.^6^ Incorporating such temporal variability into wetland emission models is essential to enhance their accuracy.

In the Iberian Peninsula, many wetlands were historically converted into wet grasslands for livestock through shallow, seasonal flooding to support grazing and mowing. However, with the decline in livestock activity—driven by reduced profitability and an aging rural population—there is now an opportunity to reintroduce natural flooding regimes and restore wetlands to their original hydrological functions. A case in point is Laguna de La Nava, where areas previously managed for livestock in Mazariegos were abandoned in 2021 following the retirement of local shepherds. The existing water management infrastructure is now being repurposed to promote renaturalization, focusing on conserving local flora and fauna under the supervision of the Junta de Castilla y León.

Considering the present study, which highlights the influence of flooding gradients, managing water levels in Mediterranean wetlands becomes a critical factor in regulating greenhouse gas (GHG) emissions and preserving ecological functions. Our findings underscore the complexity of emission patterns, driven by water saturation, depth gradients, and seasonal changes. This aligns with research suggesting that preserving natural hydroperiods, where seasonal flooding is allowed, can help reduce methane (CH_4_) emissions by supporting anoxic conditions that promote the activity of methanotrophs, which oxidize methane before it escapes into the atmosphere.

In contrast, artificial alterations to water levels can disrupt this delicate balance, often leading to increased GHG emissions—particularly methane—during dry periods, followed by significant emission pulses when reflooding occurs.^19^ Moreover, maintaining natural wet–dry cycles can optimize carbon storage and minimize phosphorus release, which would otherwise contribute to eutrophication and further destabilize wetland ecosystems.^20^ Therefore, our study emphasizes the importance of proactive water management strategies aimed at preserving these natural hydroperiods to enhance the resilience of Mediterranean wetlands and mitigate their global warming potential.

## 5 Conclusions

This study shows that greenhouse gas (GHG) emissions in Mediterranean wetlands are strongly influenced by seasonal water level variations and environmental gradients. Coastal wetlands exhibited the highest GHG emissions, while saline wetlands had the lowest. CO_2_ emissions peaked in dry zones and declined with increasing water depth, reflecting the importance of aerobic metabolism in drier conditions. In contrast, CH_4_ emissions were most variable in waterlogged transition zones, particularly in coastal wetlands during spring and summer. The findings highlight the productivity of the wetland as a key factor driving GHG production, with temperature and salinity also playing significant roles. Additionally, carbon outflow was higher in shallower zones, while CH_4_ emissions were often highest in intermediate depths that remain frequently flooded but shallow. Understanding these spatial-temporal variations along the flooding gradient is crucial for accurately modeling gas exchanges and managing wetlands for climate change mitigation and ecosystem conservation.
